# 2586. Comparison of Antimicrobial Regimens for Critically Ill Patients with Suspected or Confirmed Hospital-Acquired or Ventilator-Associated Pneumonia: A Retrospective, Cohort Study

**DOI:** 10.1093/ofid/ofad500.2201

**Published:** 2023-11-27

**Authors:** Abigail Kosharek, Kevin Betthauser, Paul Juang, Scott Micek, Dan Igles, Marin Kollef

**Affiliations:** Barnes-Jewish Hospital, Saint Louis, Missouri; La Jolla Pharmaceutical Company, Saint Louis, Missouri; St. Louis College of Pharmacy, St. Louis, Missouri; Barnes Jewish Hospital, St Louis, Missouri; Mayo Clinic, Phoenix, Arizona; Washington University, St. Louis Missouri, St Louis, Missouri

## Abstract

**Background:**

Hospital-acquired and ventilator-associated pneumonia (HAP/VAP) remain significant causes of morbidity and mortality among patients admitted to a medical facility. Consensus guidelines for the treatment of HAP/VAP recommend patients with certain risk factors be empirically treated with an anti-methicillin resistant *Staphylococcus aureus* (MRSA) and anti-pseudomonal antibiotic. The most commonly utilized anti-pseudomonal agents at BJC hospitals include cefepime, followed by piperacillin/tazobactam and meropenem. The purpose of this study is to determine if there is a difference in clinical outcomes between cefepime and the other most commonly used anti-pseudomonal antibiotics, piperacillin/tazobactam or meropenem, at these institutions for the treatment of HAP/VAP.

**Methods:**

This retrospective, multicenter cohort study included adult patients with HAP/VAP who received cefepime, meropenem, or piperacillin/tazobactam. Major exclusion criteria included patients that received antibiotics within 48 hours of hospital admission or less than 48 hours of empiric antibiotics. The primary outcome was in-hospital mortality. Secondary outcomes included change in initial antibiotic, mechanical ventilation-free days, ICU length of stay, hospital length of stay, hospital readmission, and new *C. difficile* infection.

**Results:**

Of 1,117 included patients, 1,008 patients received cefepime and 109 received meropenem or piperacillin/tazobactam. Incidence of in-hospital mortality was not observed to be significantly different among empiric Gram-negative regimens (cefepime group 19.4% vs. meropenem or piperacillin/tazobactam 22%, p=0.521. Change in initial antibiotic, ICU length of stay, and hospital length of stay were observed to be significantly higher in the cefepime group.

**Conclusion:**

We observed no difference in in-hospital mortality between cefepime and piperacillin/tazobactam or meropenem. However, there was a significantly higher incidence of change in initial antibiotic, ICU length of stay, and hospital length of stay observed in the cefepime group, though additional research is needed to corroborate these observations.

**Disclosures:**

**Kevin Betthauser, PharmD**, La Jolla Pharmaceutical Company: Advisor/Consultant|La Jolla Pharmaceutical Company: Grant/Research Support **Paul Juang, Pharm.D.**, Merck & Co: Grant/Research Support

